# Crystallization Kinetics and Structure Evolution during Annealing of Ni-Co-Mn-In Powders Obtained by Mechanical Alloying

**DOI:** 10.3390/ma16020645

**Published:** 2023-01-09

**Authors:** Edyta Matyja, Krystian Prusik, Maciej Zubko, Paweł Świec, Grzegorz Dercz, Jan Loskot

**Affiliations:** 1 Institute of Materials Engineering, University of Silesia in Katowice, 41-500 Chorzów, Poland; 2 Department of Physics, Faculty of Science, University of Hradec Králové, 500-03 Hradec Králové, Czech Republic

**Keywords:** crystallization kinetics, atomic ordering, Ni-Co-Mn-In, magnetic shape memory alloys, mechanical alloying

## Abstract

The crystallization kinetics and structure evolution during annealing of the Ni_45.5_Co_4.5_Mn_36.6_In_13.4_ (at. %) powders produced by mechanical alloying (MA) was investigated. After 70 h and 100 h of MA, the powder consisted of a mixture of amorphous and nanocrystalline body-centered cubic (bcc) phases. We observed the relaxation in the as-received powder. The relaxation temperature (T_re_) increases logarithmically with the annealing time. Annealing above 440 °C results in (1) ordering of L2_1_, (2) dissolution of the residual Ni and Mn, (3) tetragonal MnNi phase formation and (4) γ phases precipitation. The activation energies of the B2 → L2_1_ and Mn (α-Mn) → MnNi (P4/mmm) transformations were calculated.

## 1. Introduction

Ni-Mn-X (Ga, Sn, Sb, In) magnetic shape memory alloys (MSMAs) have been extensively studied in recent years. MSMAs have many advantages, such as a large magnetic-field-induced strain and stress, high working frequency, high adiabatic temperature change in the low field, rare-earth-free and non-toxic elements, large magnetoresistivity and good oxidation resistance. In addition, these materials exhibit multifunctional properties, e.g., magnetic shape memory effect, magnetocaloric, barocaloric and elastocaloric effects. MSMAs are interesting for applications, such as actuators, sensors, and micro-electro-mechanical systems (MEMS). Additionally, their unique properties play an important role in developing alternative refrigeration technology [1,2,3,4,5].

MSMAs are currently being produced as bulk, rods, thin films, etc., by conventional techniques, such as arc/induction melting, suction casting, directional solidification and magnetron sputtering. There are also several reports on MSMAs powders and nanoparticles [6,7,8,9]. Wang et al. [7] reported that Ni_2_MnGa nanoparticles undergo different structural transitions than their corresponding bulk alloy. So, it is important to determine the crystal structure, magnetostructural transformation, and properties of MSMAs nanopowders produced by various techniques. Additionally, MSMAs powders can be processed by the sintering process or additive manufacturing [10,11]. To improve ductility, Ni-Mn-based magnetic shape memory powders can also be used as reinforcement in polymer matrix composites [12,13,14,15].

MSMAs powders are usually manufactured by the following steps: (1) arc/induction melting, (2) mechanical pulverization of bulk/ribbon, and finally, (3) high-energy ball milling. It is worth underlining that ball milling is a simple, cost-effective technique suitable for industrial production [6]. The ball milling process introduced structural defects, such as vacancies, dislocations, lattice expansion, and anti-phase boundaries, which could increase the free energy and lead to amorphous phase formation [16].

Some researchers described the crystallization kinetics of amorphous Ni-Mn or Ni-Mn-Sn thin films produced by the magnetron sputtering technique [17,18,19,20]. Generally, during annealing, the phase transitions of amorphous films proceed as follows: amorphous → fcc → L1_0_. Several crystallization mechanisms have been proposed for Ni-Mn-based amorphous powders obtained by high-energy ball milling. Tian et al. [8] stated that atomic ordering in Ni_49.8_Mn_28.5_Ga_21.7_ powder results from the nucleation and growth of ordered-Heusler L2_1_ grains in the disordered bcc matrix. During heating of the ball-milled powders, they also observed the structural transition from fcc to bcc [21]. For the Ni_50_Mn_34_In_16_ powder, Sánchez-Alarcos [22] recorded a two-step transition from an “amorphous-like” phase to the B2-ordered structure, whereas Liu et al., for the same system (Ni_50_Mn_36.7_In_13.3_ powders produced by high-energy ball milling), showed the one-step transition from the amorphous phase to B2, followed by the L2_1_ ordered structure [23].

As mentioned above, in the near future, MSMAs powders can be used in additive manufacturing (AM) or sintering processing of micro to macro actuators/sensors. So, there is a need to develop an appropriate powder production method. One of the most promising is mechanical alloying. MA is a solid-state process during which the elemental powders are repeatedly cold-welded, flattened, fractured and re-welded. One can see that the final MA powder is obtained from pure elemental powders, whereas usually high-energy ball milling process starts from the bulk material of a given (appropriate) crystal structure, which is then crushed until receiving the powder of a defined size, crystal structure and properties. So, the mechanisms and processes are associated with phase formation during the MA and high-energy ball milling, and after thermal treatment of the powders, they are expected to be different. Unlike ball milling, there is a lack of investigation of structure formation and its developing mechanisms of Ni-Co-Mn-In powders obtained by the mechanical alloying technique. Although MA has been widely used for other alloys, the crystallization kinetics of the MSMAs powders obtained by MA technique has not been studied yet. So, the main aim of this paper is to investigate the crystallization kinetics of Ni-Co-Mn-In powders produced by mechanical alloying.

In this paper, we described for the first time the crystallization and atomic ordering kinetics of Ni_45.5_Co_4.5_Mn_36.6_In_13.4_ (% at.) powders obtained by mechanical alloying using a high-energy planetary ball mill.

## 2. Materials and Methods

Ni_45.5_Co_4.5_Mn_36.6_In_13.4_ (nominal compound, at. %) were produced by mechanical alloying from elemental powders of Ni (300 mesh—46 µm, 99.9% purity), Co (0.5–1.5 µm, 99.8% purity), Mn (325 mesh—44 µm, 99.5% purity), and In (325 mesh—44 µm, 99.9% purity) delivered by Strem Chemicals. MA was carried out in a high-energy planetary ball mill Fritsch Pulverisette using the tempered steel vial (80 mL) and tempered steel balls. The ball-to-powder ratio (BPR) was 17:1 and the rotation speed 250 rpm. The total milling time was 70 h and 100 h (specimens denoted as 70MA and 100MA, respectively). To avoid overheating the 10 min rest periods, every 30 min milling was applied. The milling process was carried out under an Ar atmosphere to prevent significant oxidation. The phase transformations were studied by the differential scanning calorimeter (DSC, Mettler Toledo, Columbus, OH, USA) in the temperature between −120 °C and 600 °C with a heating rate of: 10 °C/min, 20 °C/min, 30 °C/min, 40 °C/min, and 50 °C/min. The temperature of the peak was determined by the tangent method (i.e., the intersection of tangents to the slopes of the peak) (dotted lines Figure 4c). The powders were annealed in situ (in the DSC chamber) at the temperatures: 150 °C, 300 °C, 330 °C, 340 °C, 350 °C, 360 °C, 380 °C, 400 °C, 440 °C, 500 °C, and 600 °C for 2 min. The heating rate was 10 °C/min. To study the relaxation process, samples after 100 h of MA were annealed at 200 °C for 2 min, 5 min, 30 min, 60 min, 180 min, 50 h, and at 250 °C for 5 min, 30 min, and 45 min. For crystal structure and phase transitions analyses, powders were annealed at 300 °C for 10 min, 2 h, 4 h, 20 h, and 100 h. For the isothermal measurements, samples were heated up to the T_iso_ with the maximum rate (approximately 300 °C/min) and then held at that temperature for 45 min during recording.

The XRD measurements were performed using Panalytical Empyrean diffractometer with PIXcell^3D^ detector (Malvern, UK) using Cu anode (CuK_α_, λ = 1.54056 Å at 40 kV/30 mA). XRD diffraction patterns were recorded in a Bragg–Brentano geometry with angular step 0.02° 2θ and a range of 10 to 110 °2θ for as-milled powders and 10–60° 2θ for annealed powder. The (111)_L21_ superlattice diffraction peak integral intensity was used to analyze the ordering process occurring in the powders during annealing. Microstructure observations were carried out by 300 kV high-resolution transmission electron microscope JEOL JEM-3010 (Tokyo, Japan) equipped with Gatan 2k × 2k Orius™ 833 SC200D CCD camera. TEM samples were prepared by putting a drop of a powder solution (prepared in ultrasonic cleaner in isopropyl alcohol) onto a copper grid with 300 mesh.

## 3. Results and Discussion

### 3.1. Structure of As-Milled Powders

**Figure 1:**
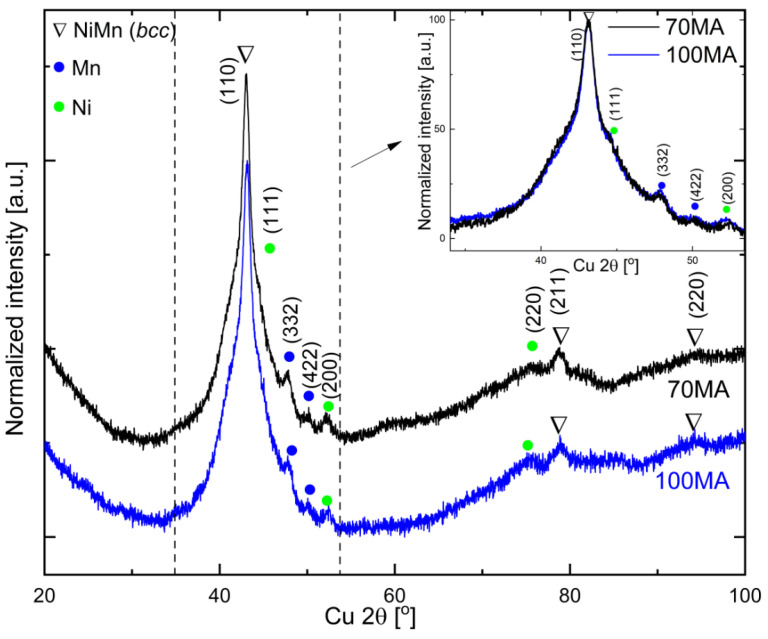
XRD patterns of as-milled powders produced by mechanical alloying for 70 h (70MA) and 100 h (100MA). Inset—superimposed XRD patters restricted to the region between dotted lines.

Figure 1 presents the XRD patterns of the as-milled Ni-Co-Mn-In powders produced by 70 h and 100 h mechanical alloying. The phase analysis revealed the disordered body-centered cubic (bcc) NiMn solid solution. There were no (h + k + l = 2n + 1) of superlattice reflections observed in the diffractogram, which suggests the formation of the disordered solid solution. Additionally, a minor fraction of pure Ni and Mn was identified in the as-milled powders. The Ni_45.5_Co_4.5_Mn_36.6_In_13.4_ solid solution formation during MA as a function of milling time (2.5–70 h) was discussed in detail in [24]. The similar values of FWHM (full width half maximum) of (110)_bcc_ peak and Ni and Mn peaks intensities indicate that prolonging milling time from 70 h to 100 h does not significantly influence the structure and phase composition of the received alloy.

**Figure 2:**
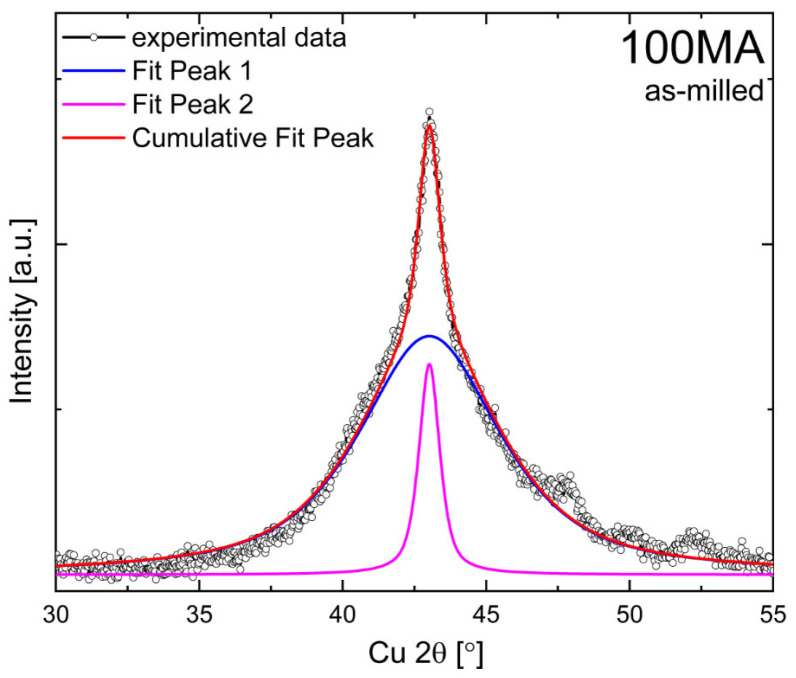
Pseudo-Voigt curves fitted to experimental XRD pattern of as-milled 100MA.

The significant broadening of the main reflection (at approximately 43° 2θ—Figure 2) indicates that the as-milled powder consists of a mixture of amorphous (or strongly refined nanocrystalline—peak 1) and bcc (peak 2) which have been identified as “the amorphous-like phase” [23,25].

**Figure 3:**
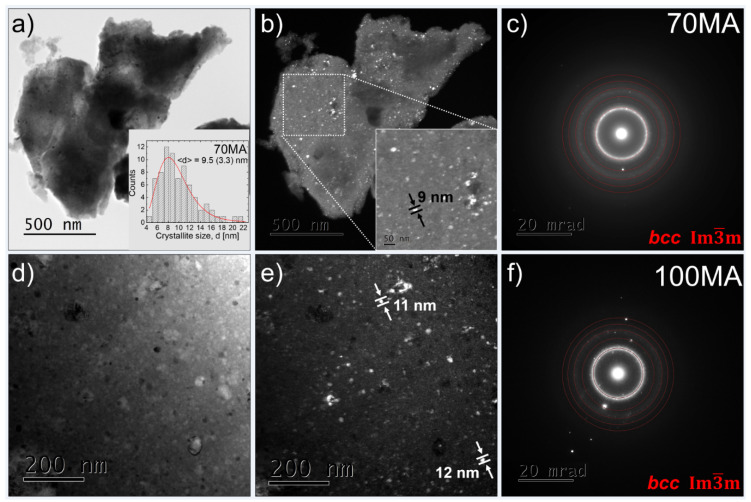
TEM results: (**a**,**d**) bright-field (BF) images, (**b**,**e**) dark-field (DF) images, (**c**,**f**) selected area electron diffraction patterns (SAEDP) of the as-milled 70MA and 100MA powders, respectively. The inset in (**a**)—histogram of the crystallite size distribution.

Figure 3 shows bright-field (BF), dark-field (DF), and selected area electron diffraction pattern (SAEDP) observed by TEM for the as-milled powder after 70 h of mechanical alloying. In the DF image (Figure 3b), one can see the nanocrystallites (white spots). The average crystallite size is 9.5 (3.3) nm (inset, Figure 3a). Additionally, the TEM results, i.e., significant broadening of the main ring in the SAEDP (Figure 3c,f), confirm the presence of the nanocrystalline/amorphous phase. It stays in good agreement with the XRD measurement for which the broadening of the (110)_bcc_ matrix reflection was observed as well.

### 3.2. DSC Measurements

#### 3.2.1. DSC Non-Isothermal Measurements

**Figure 4:**
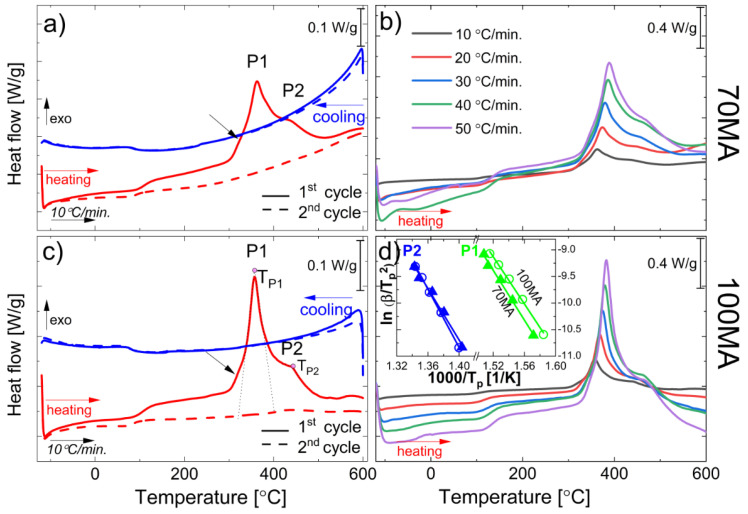
DSC curves collected for samples (**a**) 70MA and (**c**) 100MA during the 1st and 2nd cycles of cooling/heating with 10 °C/min. Peak temperature (T_p_) determined as the intersection of tangents to the peak’s slopes (dotted lines). Non-isothermal DSC curves, 1st cycle, heating rates between 105 °C/min and 50 °C/min) for: (**b**) 70MA, and (**d**) 100MA powders, inset: ln (β/T_p_^2^) vs. 1000/T_p_ of P1 and P2 peak for 70MA (triangles) and 100MA (circles) powders. Black arrows mark “bumps” in the DSC curve, referring to the crystallization of the amorphous phase.

For the DSC measurements, the as received MA powder was cooled down to −120 °C and then heated up to 600 °C (1st cycle of heating) and subsequently cooled down from 600 °C to 120 °C (1st cycle of cooling) (Figure 4a,c). The heating/cooling rate was 10 °C/min. Subsequently, the same powder sample was cycled for a second time according to the same (above) procedure. The new powder sample was used each time the DSC measurement was carried out with a different heating rate. Figure 4a,c presents DSC results of the 1st and 2nd cycle of heating the as-milled Ni_45.5_Co_4.5_Mn_36.6_In_13.4_ powders produced by 70 h and 100 h of mechanical alloying. Unlike during the 1st heating, where the exothermic peak between 300 °C and 500 °C was observed, it was not present in the 2nd heating cycle. It proves that the process to which that peak is referring is irreversible. Even after heating the powder to 600 °C (2nd cycle), the forward/reverse martensitic transformation peaks were not observed. It is worth emphasizing that the course of the cooling curves looks almost identical in both cycles. While during heating, the difference between the 1st and 2nd cycles is visible just above 100 °C. It may indicate that some other process may also happen (i.e., structural relaxation). Generally, the as-milled powder may be strongly deformed during the MA because of the high-energy ball-powder collisions. For example, it can be seen as a “jump” in the DSC curve [26,27], which for the heating of strongly deformed Ni-Ti alloy, has been ascribed to the relaxation process. Additionally, in the 2nd heating and the 1st and 2nd cooling curves at approximately 100 °C (Figure 4a,c), one can see another “jump” referring to the second-order transformation—the magnetic transformation of austenite.

#### 3.2.2. Relaxation Process

**Figure 5:**
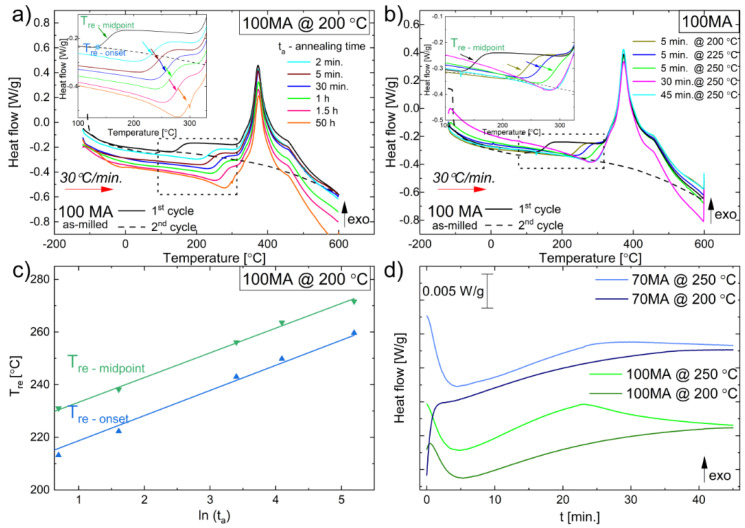
DSC heating curves recorded at 30 °C/min for 100MA as-milled powder and after annealing: (**a**) at 200 °C for 2 min, 5 min, 30 min, 1 h, 1.5 h, 50 h; (**b**) for 5 min at 200 °C, 225°C, and 250 °C and for 30 min, 45 min. Insets-magnified regions marked by dashed rectangular. Relaxation temperatures T_re_ (T_re–onset_, T_re–midpoint_) pointed out by coloured arrows. (**c**) plot of T_re_ vs. ln(t_a_), t_a_—time of annealing at 200 °C; (**d**) isothermal DSC curves at 200 °C and 250 °C for 70MA and 100MA powders.

To study the kinetics of the relaxation process, powders after 100 h of mechanical alloying were annealed at 200 °C and 250 °C for 2 min, 5 min, 30 min, 1 h, 1.5 h and 50 h. Figure 5a shows DSC heating curves collected with the 30 °C/min heating rate for 100MA samples. One can see that annealing at 200 °C causes partial relaxation. Relaxation temperature (T_re_) increases logarithmically with the annealing time (t_a_) (Figure 5a,c). The linear dependence of the T_re_ vs. ln(t_a_) is visible in Figure 5c. To initiate, in the as-milled powder, only the relaxation process, the annealing temperature should be higher than the “start” relaxation temperature (T_re_) but lower than the crystallization temperature. In our case, T_re_ ≈ 120 °C, so we chose 200 °C for annealing. The presented results show that prolonging annealing (longer than 1.5 h) at 200 °C does not significantly influence the relaxation process. Only increasing the annealing temperature above 200 °C led to the fully relaxed powder (Figure 5b). The characteristic “jump” in the DSC curves referring to the relaxation process was not observed for samples annealed at 250 °C for 30 min and 45 min. So, to ensure that the relaxation process in the as-milled powder is completed, one should anneal it at 250 °C for at least 30 min. It was also confirmed by isothermal DSC measurements at 200 °C and 250 °C for 70MA and 100MA powders (Figure 5d). One can see that “the most intense relaxation” occurs at approximately 20 min of annealing (peak in the isothermal curves at 250 °C, Figure 5d). Then, the green line in Figure 5d almost completely decayed after 40 min of annealing. It means that after 40 min of annealing at 250 °C, the 100MA powder fully relaxed. It should also be pointed out that the decrease in heat flow when the temperature increases is purely an apparatus effect, which should not be regarded as a physical effect occurring in the sample.

#### 3.2.3. Activation Energy of P1 and P2 Thermal Processes

As can be seen, several processes may contribute to the exothermic effect observed between 300 °C and 500 °C (P1 and P2 in Figure 4a,c). Additionally, at 340 °C, one can see the “bump” which arises from the crystallization of the amorphous phase (black arrows Figure 4a,c). The discussion about structural changes related to P1 and P2 is presented below. To determine the relevant kinetic parameters, such as activation energy (E_a_) of P1 and P2, the Kissinger approach was applied. According to Kissinger [28], the activation energy E_a_ means the energy barrier opposing the reaction. If the reaction proceeds at a rate varying with temperature—possesses activation energy—the position of the peak varies with the heating rate. It means that to initiate the process (to overcome the “initial” barrier), a specific energy E_a_ (activation energy) has to be supplied to the system. In a system (e.g., alloy of a given chemical composition), each process (e.g., crystallization, precipitation, phase transformation) is characterized by the separate activation energy. In this study, E_a_ is calculated based on a variation of the DSC peak temperature (T_p_) as a function of the heating rate. The DSC measurements were performed applying the following heating rates: 10 °C/min, 20 °C/min, 30 °C/min, 40 °C/min, and 50 °C/min (Figure 4b,d). The peak temperatures presented in Table 1 were determined by the intersection of tangents to the slopes of the peak. The activation energy (E_a_) was estimated from Kissinger Equation (1) as follows:

(1) $$\ln\left(\frac{\beta }{T_{p}^{2}}\right)=\ln\left(\frac{\mathrm{AR}}{E_{a}}\right)-\left(\frac{E_{a}}{{\mathrm{RT}}_{p}}\right)$$

where β—heating rate, T_p_—peak temperature, A—constant, R—gas constant.

For the 70MA and 100MA powders, the dependence of $\ln\frac{\beta }{T_{P}^{2}}$ (calculated for heating rates β = 10, 20, 30, 40 and 50 °C/min) as a function of 1/T_p_ is presented in Figure 4d (inset). Activation energy E_a_ was calculated as a linear coefficient (E_a_/R) divided by gas constant (R) (Table 2).

#### 3.2.4. DSC Isothermal Measurements

**Figure 6:**
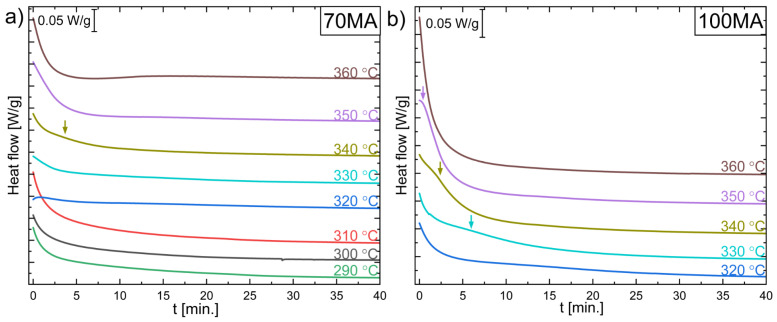
DSC isothermal curves collected at 290 °C, 300 °C, 310 °C, 320 °C, 330 °C, 340 °C, 350 °C, and 360 °C for powder after (**a**) 70 h (70MA) and (**b**) 100 h (100MA) of mechanical alloying. Coloured arrows mark the weak broad peaks.

Figure 6 shows isothermal DSC curves recorded at the temperature: 290 °C, 300 °C, 310 °C, 320 °C, 330 °C, 340 °C, 350 °C and 360 °C for 70MA and 100MA powders. The samples were initially heated up to set/measure temperatures with the maximum DSC rate (approx. 300 °C/min). Isothermal measurements are used to determine the nucleation and grain growth mechanisms of processes. By measuring the fraction of transformed material to the total enthalpy of transformation as a function of time, using the Johnson–Mehl–Avrami (JMA) model, it is possible to determine, i.e., the nucleation and grain growth rate even for the systems in which a complex multi-step crystallization process occurs [29,30,31].

As can be seen, the isothermal curves decrease monotonically. It indicates normal grain growth and cannot result from nucleation and growth transformation [30]. It is worth noticing that TEM observations of as-milled powders revealed a mixture of amorphous and bcc phases. So, it can be assumed that the annealing process causes the growth of the nanocrystalline phase grains. However, isothermal curves at 330 °C, 340 °C, and 350 °C exhibit faint peaks (arrows in Figure 6). As mentioned before, a peak in the isothermal DSC curve is connected with the nucleation and growth or abnormal grain growth process. The faint peaks at 330 °C, 340 °C, and 350 °C in isothermal curves (Figure 6) may indicate that the grains nucleate and grow on the amorphous phase.

### 3.3. Effect of Powders’ Annealing on the Phase Transformations

**Figure 7:**
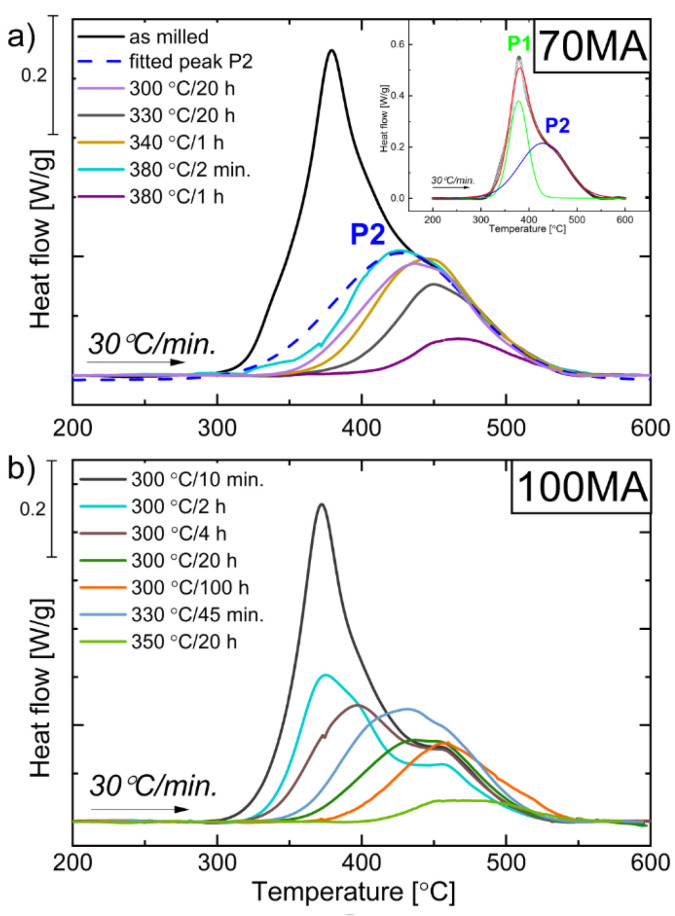
DSC curves recorded with the heating rate 30 °C/min for powders after: (**a**) 70 h of MA and annealed at 300 °C for 2 min, 330 °C for 20 h, 340 °C for 1 h, 380 °C for 2 min, and for 1 h; (**b**) 100 h of MA and annealed at 300 °C for 10 min, 2 h, 4 h, 20 h, 100 h, 330 °C for 45 min and 350 °C for 20 h. Inset—DSC line profile decomposition on P1 (green line) and P2 (blue line) processes.

**Figure 8:**
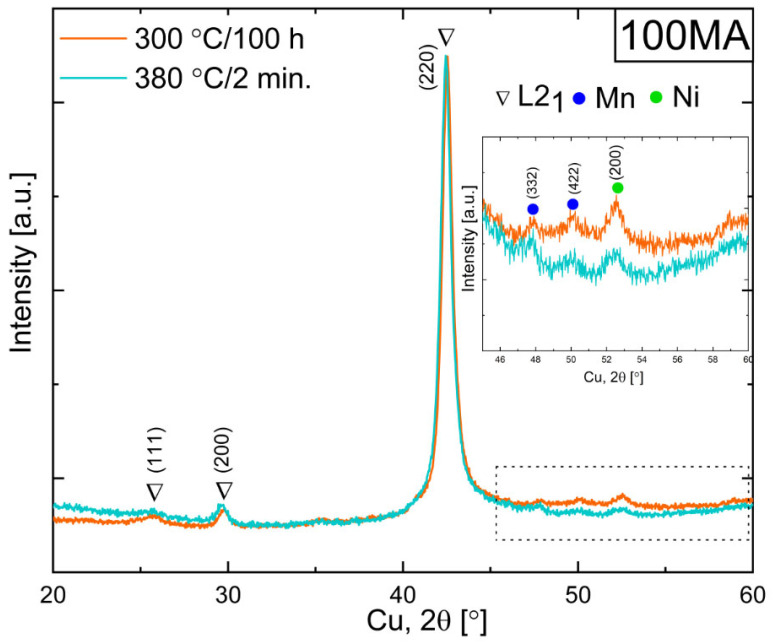
XRD patterns of powders after 100 h mechanical alloying and annealing at 300 °C for 100 h and at 380 °C for 2 min.

As mentioned above, during non-isothermal heating (Figure 4), two peaks related to P1, P2 and amorphous phase crystallization (black arrows in Figure 4) were observed. In order to explain the effects related to each peak, the appropriate heat treatment should be applied. To obtain powder for which the P1 process accomplished but P2 did not yet start, 70MA and 100MA samples were annealed at 300 °C for 10 min, 2 h, 4 h, 20 h, 100 h, at 330 °C for 45 min, 20 h, at 340 °C for 1 h, 380 °C for 2 min and 1 h. Figure 7 shows DSC heating curves recorded with a 30 °C/min heating rate. One can see that the profile (shape) of the DSC peak for the samples 70MA annealed at 380 °C for 2 min and for 100MA annealed at 300 °C for 20 h and 100 h look similar to as-milled powder P2 fitted curve (Figure 7a—inset). It means that for powders annealed at those conditions, process P1 seems to be accomplished. The XRD was applied to determine the phase composition changes during the sample annealing. In specimen 100MA annealed at 300 °C for 100 h and 380 °C for 2 min, L2_1_ Heusler-ordered phase and residual Ni, and Mn (also observed in the as-milled powders) were determined (Figure 8). One can see that annealing the powder at a lower temperature and for a longer time (at 300 °C for 100 h) allows us to obtain the powder of the same phase composition as for that annealed at a higher temperature for a shorter time (380 °C for 2 min). Additionally, during annealing, the ordering process occurs, bcc-disordered solid solution transforms into L2_1_ Heusler-ordered structure. It will be discussed in detail in Section 3.4.

### 3.4. XRD Phase Analysis of Annealed Powders

**Figure 9:**
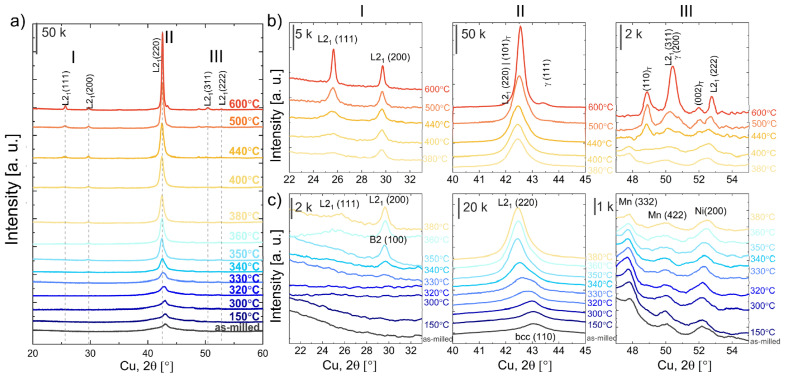
(**a**) XRD diffraction patterns of as-milled and annealed 100MA powders, 2θ ranges: (I) 21–33° 2θ, (II) 40–45° 2θ, and (III) 47–55° 2θ. For better clarity, XRD results are presented separately for temperatures: (**b**) 380–600 °C, and (**c**) from RT (as-milled) to 380 °C.

The XRD diffraction patterns of as-milled and 100MA powders annealed at 150 °C, 300 °C, 320 °C, 330 °C, 340 °C, 350 °C, 360 °C, 380 °C, 400 °C, 440 °C, 500 °C, and 600 °C for 2 min taken at room temperature are depicted in Figure 9. The XRD data presents the evolution of the phases (and/or the phase creation) in 100MA powder during the annealing. The data were analyzed in terms of: the ordering process (region I—from 21 to 32° 2θ), crystallite size (region II—from 40 to 45° 2θ) and structural changes related to the residual Mn, Ni presence (region III—from 47 to 55° 2θ). For better clarity, XRD results for samples annealed between 380 °C and 600 °C and between room temperature (as milled) and 380 °C were presented separately in Figure 9b,c.

**Figure 10:**
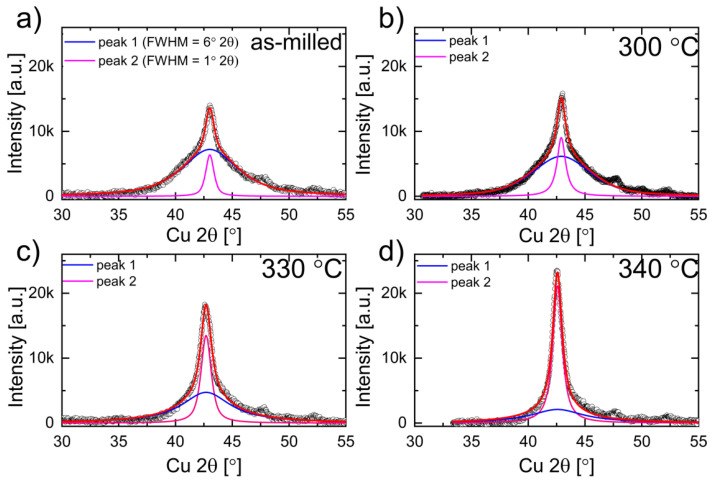
Fitted curves to experimental XRD patterns (black circles) of 100MA powders: (**a**) as-milled (RT), and annealed at: (**b**) 300 °C, (**c**) 330 °C, and (**d**) 340 °C. The fitted summary peak line profile (red line) composed of peak 1 (blue curve) which corresponds to the amorphous fraction and peak 2 (pink curve) to the crystalline fraction observed in 100MA powders.

As previously mentioned, the disordered bcc solid solution was identified in the as-milled sample. For the samples annealed above 330 °C, at approximately 30° 2θ, the (100)_B2_ peak appears (Figure 9b,c—(I)). Additionally, at approximately 25.7° 2θ, for samples annealed above 350 °C, the (111)_L21_ superlattice reflection starts to grow. One can see that intensity of the peak increases when the annealing temperature increases (Figure 9b—(I)). So, the atomic ordering occurs continuously with the temperature and time of annealing. Furthermore, the peak intensity ratio I_111_/I_200_ of the sample annealed at 600 °C is 1.66 and is equal to the theoretical value (ICDD PDF 04-017-6856). So, one may conclude that the fully ordered Heusler structure is present in the samples, annealed at 600 °C. Moreover, with the increase of annealing temperature, the intensities, angular position and profile of the (110)_bcc/B2_ ((220)_L21_) change (Figure 9—(II)). For samples annealed between 300 °C and 350 °C, the (110)_bcc/B2_ ((220)_L21_) peak shifts towards lower 2θ angles, which relates to the increase in lattice parameters of that phase. A similar effect for Ni-Mn-In powders annealed at higher temperatures (427–527 °C) obtained by ball milling was observed by Sánchez-Alarcos et al. [22]. The authors assigned the increase in lattice parameters to the relaxation and ordering of B2–L2_1_. Additionally, a significant increase in the intensity and decrease in FWHM of the (110)_bcc/B2_ peak was observed for samples annealed between 330 °C and 360 °C. To analyze that effect, the (110)_bcc/B2_ peak deconvolution and fitting were done. The peak is a sum of two different profile components: the broad blue (Figure 10, peak 1) and the narrow pink (Figure 10, peak 2), which can be assigned to amorphous and crystalline phase fractions, respectively. One can see that when the annealing temperature increases, the intensity and width of the (110)_bcc/B2_ decrease, the blue peak intensity gradually decreases and the pink peak simultaneously increases. So, during the annealing (up to 340 °C), the amorphous phase crystallizes (transforms to bcc). At the same time, the lattice strain of the crystalline fraction reduces, and the bcc/B2 crystallites start to grow. That is why the (110)_bcc/B2_ FWHM decrease and the intensity increase simultaneously.

At the higher annealing temperatures (above 440 °C), several processes/transformations occur: (1) further ordering of L2_1_ and crystallite growth (as mentioned above); and dissolution or formation of the minor phases as: (2) the dissolution of the residual Ni and Mn, (3) MnNi (tetragonal) formation and (4) γ phases formation. During the annealing of the 100MA sample, between 150 °C and 400 °C, no changes in the intensity and profile of the 332_Mn_, 422_Mn_, and 200_Ni_ lines were observed (Figure 9—III). Whereas for powders annealed at 440 °C (and higher), Mn and Ni lines gradually disappear while new peaks in the same region of 2θ (at 48.90° 2θ and 51.96° 2θ) related to the MnNi tetragonal phase creation appear (space group P4/mmm, lattice parameters *a*_0_ = 2.62 Å *c*_0_ = 3.52 Å ICDD PDF 04-006-8085). Additionally, for powder annealed at 600 °C at 43.32° 2θ, one can see the (200)_γ_ the fcc–face-centred cubic γ phase peak (*a*_0_ = 3.606 Å, ICDD PDF 04-017-5179) (Figure 9b—II). The γ probably nucleates on the residual Ni (present in the as-milled powder), which did not dissolve in the bcc parent phase during mechanical alloying and did not transform to the NiMn-tetragonal phase. As annealing procced Co and Mn migrate into the Ni, growing the Ni-based fcc-solid-solution (γ).

Taking the above into account, during the annealing of the Ni_45.5_Co_4.5_Mn_36.6_In_13.4_ powders obtained by mechanical alloying, the phase transformations occur in the following order:

$$\mathrm{amorphous}\overset{300-340\;{}^{\circ}C}{\to }\mathrm{disordered}\;\mathrm{bcc}\overset{340\;{}^{\circ}C}{\to }B2\;\left(\mathrm{ordered}\;\mathrm{bcc}\right)\overset{360\;{}^{\circ}C}{\to }L2_{1}\;\left(\mathrm{ordered}\;\mathrm{Heusler}\right)$$

$$\mathrm{Ni},\mathrm{Mn}\;\left(\mathrm{residual}\right)\overset{440\;{}^{\circ}C}{\to }\mathrm{NiMn}\;\left(\mathrm{tetragonal}\;\mathrm{phase}\right)$$

$$\mathrm{Ni},\mathrm{Mn}\;\left(\mathrm{residual}\right)\overset{600\;{}^{\circ}C}{\to }\;\gamma \;\mathrm{phase}$$

It should be clear that the as-milled powder consists of the mixture of the disordered bcc parent phase, the amorphous phase and the residual Mn and Ni. During the annealing between 300 °C and 340 °C, the amorphous phase transforms into the disordered bcc. At approximately 340 °C, the bcc to B2 transformation starts and continues up to 360 °C, where the B2 to ordered L2_1_ Heusler occurs. The powder annealed between 360 °C and 600 °C consists of the partially-ordered Heusler compound. The fully ordered L2_1_ Heusler structure is observed for powders annealed at 600 °C. Due to the atomic ordering being a time-dependent process, holding a specimen long enough, even just above 360 °C, one may also expect to obtain the fully ordered L2_1_ Heusler structure.

Depending on the chemical composition, the manufacturing and the thermal treatment, the transformations in NiMn-based alloys may occur in a different order. Table 3 presents the phase transformation activation energies of the Ni-Mn-Z (In, Ga) alloys (powders, thin films) consisting of amorphous or “amorphous-like” phases. In NiMn films obtained by magnetron sputtering, usually, the amorphous (or “amorphous-like”) phase transforms into fcc phase followed by fcc → L1_0_ [18,19]. However, powders produced by high-energy grinding reveal different sequences of phase transitions during annealing. Sánchez-Alarcos et al. [22] in Ni_50_Mn_34_In_16_ powders registered a two-step transformation from amorphous to B2 structure. In contrast, Tian et al. [21] fcc into bcc transition in Ni_49.8_Mn_28.5_Ga_21.7_ powders were observed. They also [8] found that above 360 °C, small domains of the ordered L2_1_ Heusler phase were formed in the disordered bcc matrix. The volume fraction of the L2_1_ phase increased with the annealing temperature increases. Considering the above, one can expect similar structural evolution during the annealing of the NiCoMnIn powders produced by mechanical alloying (this work). We observed nucleation and growth of the ordered L2_1_ structure when the annealing temperature increased. It was confirmed by the increase of the superlattice reflection intensity ratio (I_(111)_/I_(200)_) (Figure 9 b). Additionally, during the annealing above 440 °C, the tetragonal phase can be formed. It is due to the lattice distortion of the parent phase and L2_1_ growth. Liu et al. [23] observed a tetragonally distorted Heusler phase in Ni_50_Mn_36.7_In_13.3_ powders annealed for 30 min at 411 °C (684 K). The activation energies (E_a_) determined for studied Ni_45.5_Co_4.5_Mn_36.6_In_13.4_ MA alloys are similar to those observed for other magnetic shape memory powders obtained by high-energy-ball-milling (e.g., Ni-Mn-Ga and Ni-Mn-In) and slightly higher than for NiMn-based films obtained by magnetron sputtering. Despite that, for different alloys, E_a_ values cannot be directly compared (especially for the different transformations), it may give a rough idea about the expected kinetics parameters of the processes that occur in the sample.

## 4. Summary and Conclusions

In this work, the crystallization kinetics and structure evolution of the thermally treated Ni_45.5_Co_4.5_Mn_36.6_In_13.4_ powders obtained by mechanical alloying (MA) were analyzed. We observed that low-temperature annealing led to the relaxation of the stresses accumulated in the powder during the prolonged milling (70 h). Although the process starts at 100 °C, the powder to be fully relaxed, should be annealed at 250 °C for at least 30 min. The relaxation temperature (T_re_) logarithmically increases with the annealing time. After 70 h and 100 h of milling, the powder consisted of a mixture of the amorphous phase, nanocrystalline body-centred cubic (bcc) disordered solid solution and minor fractions of non-dissolved Ni and Mn. During annealing between 300 °C and 340 °C, the amorphous phase transforms into the disordered bcc. At 340 °C, the bcc to B2 transformation starts and continues up to 360 °C, where the B2 to L2_1_ Heusler transition occurs. At the higher annealing temperatures (above 440 °C), the following processes/transformations occur:

(1) Further ordering of L2_1_ phase and its growth (the fully ordered L2_1_ Heusler structure was observed for powder annealed at 600 °C);
(2) Dissolution of the residual Ni and Mn;
(3) MnNi (P4/mmm) phase formation;
(4) γ (fcc) phases precipitation.

Using the Kissinger approach, the activation energies (E_a_) of the transition processes B2 → L2_1_ and Mn (α-Mn) → MnNi (P4/mmm) were calculated as 185(6) kJ/mol and 228(3) kJ/mol, respectively.

Gaining knowledge about crystallization kinetics, structures of the phases and transformation sequence during thermal treatment of the mechanically alloyed powders is advantageous and may help to design the materials for future applications.

## Figures and Tables

**Table 1:** T_P1_, T_P2_—peak temperatures of P1 and P2 process observed in non-isothermal DSC heating curves recorded with 10 °C/min, 20 °C/min, 30 °C/min, 40 °C/min, and 50 °C/min heating rate (β).

β [°C/min]	70MA T_P1_ [°C]	70MA T_P2_ [°C]	100MA T_P1_ [°C]	100MA T_P2_ [°C]
10	363.2	439.3	358.0	441.3
20	374.2	451.2	368.9	453.2
30	380.7	459.2	375.9	461.2
40	387.6	468.3	381.7	466.3
50	389.9	471.5	386.6	471.0

**Table 2:** The activation energies of the processes, P1 and P2, observed in DSC non-isothermal heating curves. Calculation based on Kissinger equation.

Sample	E_a_ [kJ/mol] P1	E_a_ [kJ/mol] P2
70MA	195 (8)	202 (12)
100MA	185 (6)	228 (3)

**Table 3:** The activation energies (E_a_) and transformation sequences observed in MnMn-based alloys obtained by different techniques.

Alloy Composition, State (Form)	Manufacturing Method	Activation Energy E_a_	Process	Ref.
Ni_45.5_Co_4.5_Mn_36.6_In_13.4_ powder	Mechanical alloying (100MA)	185(6) kJ/mol 228(3) kJ/mol	300 °C to 340 °C amorphous phase → bcc 340 °C: bcc → B2 360 °C: (P1) B2 → L2_1_ (P1) 440 °C: (P2) Mn (α-Mn) → MnNi (P4/mmm)	This work
Ni_50_Mn_34_In_16_ powder	high-energy ball milling	164.0 kJ/mol (1) 173.7 kJ/mol (2)	two-step (1) (2) transformation amorphous-like phase → B2-ordered structure	[22]
Ni_49.8_Mn_28.5_Ga_21.7_ powders	high-energy ball milling	209(8) kJ/mol	fcc → bcc	[21]
Ni_50_Mn_36.7_In_13.3_ powder	high-energy ball milling	No data	523 K (250 °C) amorphous like phase → B2 annealing at 684 K (411 °C) 30 min B2 → L2_1_ with tetragonal distortion	[23]
Ni_49.8_Mn_28.5_Ga_21.7_ powders	high-energy ball milling	No data	(305 °C) fct → bcc (fct—face-centered tetragonal) (360 °C) L2_1_ nucleation and growth with increased annealing temperature (sample annealed at 800 °C—full Heusler structure)	[8]
NiMn films ^1^	DC magnetron sputtering	139 kJ/mol	disordered fcc → L1_0_ (L1_0_—tetragonal)	[19]
NiMn films ^1^	DC magnetron sputtering	121 kJ/mol 140 kJ/mol	(1) Amorphous → fcc (2) fcc → L1_0_	[18]
NiMn films ^1^	DC magnetron sputtering	No data	(1) 160 °C MnO formation (2) 320 °C—amorphous like phase → fcc (3) 360 °C fcc → L1_0_ (4) 440 °C grain growth	[20]

^1^ Obtained from elementary targets.

## Data Availability

Not applicable.
